# Systemic Loss of Chromosome Y Associates With Age‐Related Ophthalmic Diseases in Middle‐Aged Men: A Longitudinal Multi‐Omic Study

**DOI:** 10.1111/acel.70775

**Published:** 2026-10-18

**Authors:** Tianhao Chen, Zhaoyu Xiang, Zhe Zhang, Bingqing Sun, Xuan Ding, Liyin Wang, Yunqian Yao, Zhuotong Wu, Siyang Liu, Yuan Lei, Xingtao Zhou, Jing Zhao

**Affiliations:** ^1^ Eye Institute and Department of Ophthalmology Eye & ENT Hospital, Fudan University Shanghai China; ^2^ NHC Key Laboratory of Myopia and Related Eye Diseases Chinese Academy of Medical Sciences Shanghai China; ^3^ Shanghai Research Center of Ophthalmology and Optometry Eye & ENT Hospital, Fudan University Shanghai China; ^4^ Shanghai Engineering Research Center of Laser and Autostereoscopic 3D for Vision Care Eye & ENT Hospital, Fudan University Shanghai China; ^5^ Shanghai Key Laboratory of Gene Editing and Cell Therapy for Rare Diseases Fudan University Shanghai China; ^6^ Shanghai Key Laboratory of Visual Impairment and Restoration Shanghai China

**Keywords:** age‐related ophthalmic disease, AMD, loss of chromosome Y, myeloid cells, proteomics, single‐cell RNA sequencing, somatic mutation

## Abstract

Maintaining health during global population aging represents a critical challenge, where mosaic loss of chromosome Y (LOY) in the hematopoietic system surfaces as a prevalent somatic mutation in aging men. However, its longitudinal impact on sensory organ degeneration remains insufficiently characterized. Analyzing 223,051 male participants from the UK Biobank, we demonstrated that LOY uniquely and independently predicted an increased risk of incident age‐related macular degeneration (AMD) (Hazard Ratio = 0.611, 95% Confidence Interval: 0.397–0.942). Restricted cubic splines identified a critical risk escalation threshold at −0.17 for median log R ratio. Over a 15‐year preclinical window, Olink plasma proteomics identified 41 prioritized proteins demonstrating divergent nonlinear kinetic profiles leading up to clinical diagnosis; notably, the elastin‐degrading matrix metalloproteinase‐12 deviated significantly 12 years prior to endpoint. Integrating single‐cell RNA sequencing datasets of peripheral blood and choroidal tissues validated that CD16^+^ circulatory monocytes and resident macrophages exhibited peak LOY frequencies. Furthermore, Y‐deficient myeloid cells manifested a pro‐inflammatory phenotype with cell‐type‐specific upregulation of *IFI30* and *CKB*, bridging single‐cell transcriptional activation with systemic proteomic dysregulation. Causal mediation analysis confirmed that peripheral monocyte counts significantly mediated the LOY‐associated degenerative risk. Utilizing these prioritized proteomic signatures, a LightGBM machine learning framework achieved robust predictive performance for 5‐year incident risk (AUC = 0.734). Collectively, these findings establish a distinct somatic mutation‐immune‐retinal axis, positioning systemic clonal mosaicism not merely as a marker of biological senescence but as an upstream driver of site‐specific organ degeneration, offering novel strategies for early risk stratification and precision intervention in aging‐related diseases.

## Graphical abstract

This longitudinal, multi‐omic study of 223,051 men identifies systemic mosaic loss of chromosome Y (LOY) as an independent risk factor for age‐related macular degeneration (AMD). By integrating prospective plasma proteomics and single‐cell transcriptomics, we unveil a distinct somatic mutation‐immune‐retinal axis wherein Y‐deficient myeloid cells drive early proteomic dysregulation and local macrophage remodeling, positioning clonal hematopoietic mosaicism as a critical systemic driver of site‐specific organ senescence.

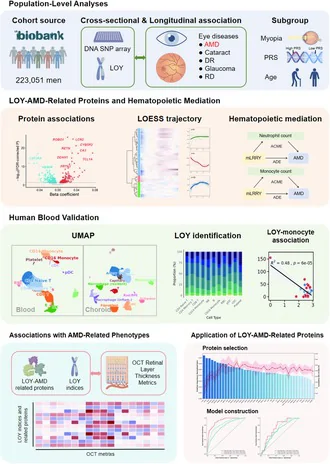

## 1 Introduction

Against the global backdrop of population aging, preserving visual function has emerged as a paramount challenge in clinical ophthalmology (GBD 2019 Blindness and Vision Impairment Collaborators and Vision Loss Expert Group of the Global Burden of Disease Study 2021). The incidence of various vision‐threatening conditions escalates significantly with age, encompassing cataract characterized by lenticular opacity, glaucoma associated with optic nerve damage, diabetic retinopathy linked to metabolic dysfunction, retinal detachment, and age‐related macular degeneration (AMD). Despite their diverse anatomical localizations and clinical manifestations, these disorders often share convergent pathophysiological underpinnings, such as chronic systemic inflammation (Huang et al. 2026; Khan et al. 2023) and biological senescence (Gao et al. 2025; Chen, Cao, et al. 2025; Chi et al. 2026). This shared etiology suggests that degenerative shifts within the visual system may not represent isolated local phenomena but rather ocular manifestations of a broader systemic collapse in biological homeostasis.

While large‐scale genome‐wide association studies (GWAS) have identified numerous germline genetic liabilities, the mechanisms by which stochastic environmental factors and somatic mutations contribute to these age‐related visual impairments via the ocular‐systemic axis remain insufficiently understood. Loss of chromosome Y (LOY), the most prevalent somatic mutation in the hematopoietic system of aging men, has recently surfaced as a potent biomarker for various age‐associated morbidities (Sato et al. 2026; Weng et al. 2024; Wang, Chen, et al. 2025; Su et al. 2025). Beyond its role as a simple indicator of biological aging, LOY has been mechanistically linked to clonal hematopoiesis (Dawoud et al. 2023) and an increased risk of non‐hematological conditions, including cardiovascular diseases (Sano et al. 2022; Hussain et al. 2026) and cancer (Abdel‐Hafiz et al. 2023; Thompson et al. 2019). Given that LOY appears to modulate leukocyte function and systemic inflammatory profiles (Sano et al. 2022; Abdel‐Hafiz et al. 2023), it presents a compelling candidate for exploring the link between systemic somatic mutations and site‐specific ocular degeneration. However, prospective evidence establishing LOY as a robust risk factor for age‐related ophthalmic diseases, particularly its impact on retinal microarchitecture and its mediation via the myeloid lineage, remains absent in current ophthalmic literature.

The integration of high‐throughput proteomics with single‐cell transcriptomics offers a transformative opportunity to bridge the gap between systemic mosaicism and localized ocular pathology. By utilizing the Olink Explore platform and single‐cell RNA sequencing, it is now possible to map the temporal evolution of circulating proteins and their cellular origins in relation to both LOY status and future disease manifestation. This multi‐omics approach is essential for identifying the specific molecular drivers—such as those involved in complement overactivation and myeloid‐driven tissue remodeling—that characterize the LOY‐ocular disease axis.

By integrating a large‐scale prospective cohort with multi‐omic evidence, we unveil the longitudinal associations between LOY and specific aging‐related ocular phenotypes alongside their underlying biological mechanisms. Utilizing single‐cell transcriptomics and plasma proteomic trajectory modeling, we identified monocyte‐associated systemic inflammation as a critical nexus linking LOY to alterations in the retinal microenvironment and developed a high‐performance proteomic prognostic model. These findings not only elucidate the driving role of somatic mosaicism in sensory degradation but also provide pivotal strategies for the early warning and precision management of age‐related ophthalmic diseases.

## 2 Methods

### 2.1 Data Source and Study Population

The UK Biobank (UKB) is a comprehensive, large‐scale prospective cohort study comprising over 500,000 participants aged 40–69 years at recruitment (Foster et al. 2023). Participants were recruited between 2006 and 2010 across 22 assessment centers distributed throughout the United Kingdom. These centers are integrated with the National Health Service (NHS), facilitating systematic and longitudinal follow‐up through linked electronic health records (Sudlow et al. 2015). Ethical approval for the UKB was granted by the National Information Governance Board for Health and Social Care and the NHS North West Multicenter Research Ethics Committee. All participants provided informed consent via electronic signature prior to enrollment. This study was conducted under UKB Application Number 105765 and adheres to the Strengthening the Reporting of Observational Studies in Epidemiology (STROBE) guidelines for cohort studies. The study population was prospectively derived from the UKB database, with recruitment occurring during routine outpatient visits at the designated assessment centers.

### 2.2 Estimation of LOY

For the identified male study population, mosaicism of chromosome Y was characterized using log R ratio (LRR, corresponding to the probe intensity Log2 R Ratio [L2R] values derived from the Affymetrix arrays and widely termed LRR as an established field convention) and B‐allele frequency (BAF) metrics derived from the UK Biobank genotyping arrays. Specifically, LOY status was determined by analyzing the median LRR (mLRRY) across 691 SNP probes situated within the male‐specific region of the Y chromosome (MSY; genomic coordinates ChrY: 2,658,271–28,767,492, GRCh37/hg19) (Forsberg et al. 2014). As LRR values serve as normalized indicators of signal intensity reflecting relative DNA copy number, deviations from the expected baseline were utilized to quantify genomic loss. Following the analytical framework for genotyping noise established by Forsberg et al. (2014), we defined LOY carriers as individuals whose mLRRY fell below the lower limit of the 99% confidence interval (CI). Based on this statistical threshold, a cutoff value of −0.096 was applied to identify carriers, which corresponds to an estimated cellular mosaicism fraction of 12.161%, calculated via the formula: LOY (%) = 100 × (1–2^2×mLRRY^) (Su et al. 2025). Conversely, participants with an mLRRY exceeding 0.15 were excluded from the analysis to eliminate potential confounding from other sex chromosome aneuploidies, such as XYY syndrome (Su et al. 2025). Participants with values between these thresholds were categorized as noncarriers. It should be noted that these three representations of LOY—continuous mLRRY, estimated cell percentage, and binary status—are derived from the same underlying metric and are thus highly correlated rather than mutually independent; they were evaluated in parallel to ensure the robustness and consistency of our findings across different analytical thresholds.

### 2.3 Definition of Clinical Outcomes

Clinical endpoints within the UKB cohort were ascertained through the integration of Hospital Episode Statistics (HES) data. Disease incidence was defined based on the first occurrence of the corresponding three‐digit International Classification of Diseases, 10th Revision (ICD‐10) codes recorded in inpatient electronic health records (data fields 41,270 and 41,280). The follow‐up period for each participant was calculated from the date of baseline assessment until the earliest occurrence of the following events: the initial diagnosis of the target disease, death, or the study census date (November 2023), based on the most recent available inpatient data. Guided by the ICD‐10 classification, we prioritized five major ophthalmic conditions as the primary study outcomes: cataract, glaucoma, diabetic retinopathy, retinal detachment, and AMD (Table S1).

### 2.4 Proteomics Profiling and Quality Control

Plasma proteomics data were derived from a subset of the UKB cohort, involving baseline samples from over 50,000 participants (Sun, Chiou, et al. 2023). The sample processing workflow involved blood collection into EDTA tubes, followed by centrifugation (2500 g for 10 min at 4°C) to isolate plasma, which was subsequently cryopreserved at −80°C. Proteomic quantification was performed using the Olink Explore Proximity Extension Assay (PEA) platform. This high‐throughput methodology measured 2923 protein analytes (representing 2941 unique proteins) across multiple specialized panels.

Protein abundance is reported as Normalized Protein eXpression (NPX) values, a relative quantification on a log2 scale. According to the UKB protocol, these data had already undergone rigorous quality control (Eldjarn et al. 2023)—maintaining acceptable inter‐ and intra‐assay coefficients of variation (typically < 20% and < 10%, respectively)—along with extension control normalization and bridge‐normalization to minimize technical variation and batch effects. We directly utilized these officially provided NPX values without further raw data transformation.

### 2.5 Hematological Indices and Peripheral Blood Profiling

Baseline hematological profiling was conducted using venous blood samples analyzed via the automated Coulter LH 750 System (Beckman Coulter, Brea, CA) according to standardized UKB protocols. The analysis integrated comprehensive indices across multiple hematopoietic lineages, including total white blood cell (WBC) counts and absolute differential counts for leukocyte subtypes (neutrophils, lymphocytes, monocytes, eosinophils, and basophils), erythroid parameters (erythrocyte counts, hemoglobin concentration, mean corpuscular volume [MCV], and immature reticulocyte fraction [IRF]), and total thrombocyte quantification (Table S2). All procedures adhered to rigorous internal quality control standards to ensure analytical reliability and reproducibility.

### 2.6 Polygenic Risk Score (PRS) Construction

Individual genetic predisposition to AMD was quantified using the standardized PRS provided by the UK Biobank (Data field: 26204; Table S2) (Thompson et al. 2024). This score was derived from summary statistics of an independent, large‐scale GWAS meta‐analysis. The international large‐scale AMD GWAS underlying the official UKB PRS was obtained from the International Age‐related Macular Degeneration Genomics Consortium (IAMDGC) (Fritsche et al. 2016). The computational framework utilized a Bayesian methodology, wherein posterior effect sizes for each variant were aggregated and weighted by individual allele dosages. To ensure cross‐ancestry comparability and mitigate potential biases, the PRS was standardized by the standard deviation (SD) of a representative ancestry group from the 1000 Genomes Project reference panel. This analytical procedure yielded a centered, unit‐variance standardized PRS, facilitating its implementation as a robust metric for genetic liability within the UKB cohort.

### 2.7 Refractometry and Assessment of Myopia

Refractive status was assessed by trained technicians using the Tomey RC‐5000 autorefractor (UKB Data Category 100014; Table S2) (Chua et al. 2019). To represent the baseline refractive profile of each participant, measurements from the right eye were utilized for downstream analyses. The spherical equivalent (SE) was calculated as the sum of the spherical power and half of the cylindrical power. To ensure data integrity and minimize the influence of spurious measurements, individuals with SE values exceeding three SD from the cohort mean were excluded. Myopia was clinically defined as a SE below −0.75 diopters (D).

### 2.8 Optical Coherence Tomography and Retinal Morphometry

Optical coherence tomography (OCT) imaging data utilized in this study were obtained from the UK Biobank database. Macular spectral‐domain OCT (SD‐OCT) was conducted using the Topcon 3D OCT‐1000 Mark II system under mesopic conditions without prior mydriasis. The acquisition protocol utilized a standardized 6 × 6 mm macular cube scan, consisting of a 128 (horizontal) × 512 (vertical) A‐scan matrix. Automated segmentation and layer‐specific thickness quantification were performed using the validated Topcon Advanced Boundary Segmentation (TABS) algorithm (Yang et al. 2010).

Our investigation systematically evaluated 40 primary OCT‐derived morphometric indices generated by the segmentation pipeline. These parameters encompassed global macular thickness and volume, alongside the average thickness of discrete retinal layers extending from the inner limiting membrane (ILM) to the RPE. Specifically, we analyzed the retinal nerve fiber layer (RNFL), the ganglion cell–inner plexiform layer (GC‐IPL), and the inner nuclear layer (INL). Sublayer thickness metrics defined by the TABS algorithm—including the external limiting membrane to inner segment/outer segment junction (ELM‐ISOS), INL to ELM, INL to RPE, and ISOS to RPE—were also assessed. Additionally, RPE thickness was quantified globally and within the central, inner, and outer subfields. Retinal thickness measurements were further localized across the nine standard Early Treatment Diabetic Retinopathy Study (ETDRS) grid sectors (central, inner, and outer regions). A detailed inventory and technical definitions of all 40 parameters are presented in Table S2.

Rigorous image quality control was applied following established protocols (Zekavat et al. 2022). Scans were discarded if the manufacturer‐specific quality index was below 40 or if the ILM indicator fell within the lowest decile of the study population. To ensure data integrity, thickness measurements exceeding 2.5 SD from the cohort mean were excluded. Further clinical exclusion criteria were implemented to minimize confounding effects (Peng et al. 2026): best‐corrected visual acuity (BCVA) > 0.1 logMAR, spherical equivalent refractive error beyond ±6.0 D, and intraocular pressure (IOP) outside the 5–21 mmHg range. Individuals with a history of glaucoma, AMD, significant retinal pathologies, or major neurodegenerative conditions were excluded. For participants where both eyes fulfilled the eligibility requirements, one eye was selected via random allocation; otherwise, the single eligible eye was utilized for analysis.

### 2.9 Covariate Assessment

A comprehensive set of covariates, identified or hypothesized to be associated with aging phenotypes or retinal architecture, was incorporated into the analytical models. Socio‐demographic variables included age at baseline assessment, self‐reported ethnicity (categorized as White or non‐White), the Townsend Deprivation Index, and educational attainment (dichotomized into university degree or other). Lifestyle and anthropometric factors encompassed body mass index (BMI, kg/m^2^), smoking status (never, former, or current), and alcohol consumption frequency (never, former, or current). Medical history, specifically physician‐diagnosed hypertension and diabetes mellitus (yes or no), was also ascertained. Detailed data field identifiers for all evaluated covariates are provided in Table S2.

### 2.10 Protein Trajectory Modeling and Hierarchical Clustering

Temporal dynamics of plasma proteins relative to disease manifestation were characterized as a function of the follow‐up duration. To establish a robust baseline, we performed a 1:10 case–control matching using the “MatchIt” R package, aligning individuals who developed cases with healthy controls based on the full suite of covariates employed in the survival models (age, ethnicity, education, socioeconomic status, BMI, and tobacco and alcohol utilization). Protein concentrations were standardized into *Z*‐scores using the mean and SD derived from the matched control cohort at corresponding time intervals.

The longitudinal relationship between individual protein fluctuations and disease progression was subsequently modeled using locally estimated scatterplot smoothing (LOESS) regression (Lehallier et al. 2019; Piehl et al. 2022). Proteins exhibiting a peak deviation exceeding a *Z*‐score threshold of 0.25 were prioritized for further analysis (Zhang et al. 2025). To categorize the identified trajectories, we employed an unsupervised hierarchical clustering framework via the hclust function. The Euclidean distance was utilized to quantify pairwise dissimilarities between LOESS‐predicted trends, and Ward's minimum variance method was applied to partition the proteins into three distinct clusters reflecting divergent temporal patterns.

### 2.11 Functional Enrichment Analysis

To characterize the biological functional landscape of the identified disease‐associated proteins, we conducted enrichment analyses using the Gene Ontology (GO) frameworks. These analyses were facilitated through the Database for Annotation, Visualization and Integrated Discovery (DAVID; versions 6.3 and 7.9). Statistical significance for enriched biological processes, molecular functions, and cellular components was determined using an FDR‐corrected *p* value threshold of < 0.05.

### 2.12 Mendelian Randomization

To assess the causal effects of genetically predicted monocyte immunophenotypes on AMD, a two‐sample Mendelian randomization (MR) framework was performed. Specifically, genetic summary statistics for monocyte traits were obtained from previous large‐scale genome‐wide association studies (GWAS) (UK Biobank Whole‐Genome Sequencing Consortium 2025; Dennis et al. 2021; Chen et al. 2020), while outcome data for AMD were sourced from large cohorts including FinnGen and the Million Veteran Program (MVP) (Verma et al. 2024). In the original GWAS, both exposure and outcome data sources adjusted for potential confounders such as age, sex, and ancestry‐informative principal components to account for population stratification, ensuring that a comparable set of covariates was utilized across the two samples. Instrumental variables (Single Nucleotide Polymorphisms [SNPs]) for monocyte traits were extracted at genome‐wide significance (*p* < 5 × 10^−8^) and clumped for independence (*r* ^2^ < 0.001 within a 10,000 kb window). Weak instruments were filtered out by restricting variants to an *F*‐statistic ≥ 10 (*F* = β^2^/SE^2^). To ensure robustness, a three‐tier data filtering strategy was implemented: Set1_Original (baseline instrument pool), Set2_NoRadial (excluding outliers via IVW RadialMR at *α* = 0.05), and Set3_NoPalin (excluding ambiguous palindromic SNPs). Primary causal estimates were calculated using the inverse variance weighted (IVW) method, supplemented by MR‐Egger regression, Weighted median, Simple mode, and Weighted mode. Multiple testing correction was performed using the FDR. All analyses were performed using R software (version 4.3.2) utilizing the TwoSampleMR package (version 0.5.8).

### 2.13 Machine Learning Framework for Risk Prediction

We developed a two‐stage machine learning framework to predict the long‐term incidence of disease utilizing baseline plasma proteomic profiles. The study population was randomly partitioned into a discovery cohort (80%) and an independent validation cohort (20%) using stratified sampling to maintain consistent event‐to‐non‐event ratios across subsets. In the initial discovery phase, candidate proteins were ranked by their predictive contribution using the gain‐based importance metric within a LightGBM architecture (Ke et al. 2017). Subsequently, a progressive forward selection (PFS) strategy was implemented, where proteins were iteratively incorporated into the model based on their importance ranking. The cumulative model performance was evaluated via 5‐fold cross‐validation (CV), and the optimal protein panel was determined by identifying the plateau in the area under the receiver operating characteristic curve (AUC), balancing parsimony with predictive robustness.

Hyperparameter optimization for the finalized protein subset was conducted through a large‐scale randomized search (*n* = 1000 iterations), yielding a robust configuration (e.g., max_depth = 12, n_estimators = 685, and num_leaves = 12) to maximize fitting precision (Pedregosa et al. 2011). To assess temporal predictive efficacy, sensitivity analyses were performed for risk windows at 5, 10, and 15 years post‐baseline.

### 2.14 Single‐Cell Transcriptomic Validation

#### 2.14.1 Sample Acquisition and Data Pre‐Processing

Publicly available single‐cell RNA sequencing (scRNA‐seq) datasets of peripheral blood mononuclear cells (PBMCs) and choroidal tissues were obtained for male patients with AMD (*n* = 18) and matched male controls (*n* = 9) (Lin et al. 2024). Downstream analyses were conducted using the Seurat (v5) framework. To ensure high‐quality transcriptomic profiles, stringent QC filters were applied: cells were excluded if they contained fewer than 800 or more than 3500 detected genes or exhibited a mitochondrial gene fraction exceeding 10%.

#### 2.14.2 Data Integration and Cell‐Type Annotation

Following library‐size normalization and the identification of highly variable features, the Harmony algorithm was employed to integrate the PBMC datasets, effectively mitigating interindividual variation and potential batch effects. Dimensionality reduction and unsupervised clustering were performed using Uniform Manifold Approximation and Projection (UMAP) based on the Harmony‐corrected principal components. Major cell lineages were manually annotated according to established canonical marker genes derived from existing literature.

#### 2.14.3 Quantification of Single‐Cell LOY Scores

To assess Y‐chromosome loss at single‐cell resolution, we developed a LOY‐score metric based on a core gene set within the MSY (Wang, Chen, et al. 2025). This panel comprised 10 Y‐specific genes: RPS4Y1, EIF1AY, DDX3Y, UTY, KDM5D, USP9Y, ZFY, NLGN4Y, PRKY, and TMSB4Y. The LOY‐score for an individual cell was defined as the cumulative count of these MSY genes with an expression level > 0. Cells with a LOY‐score of 0 were categorized as candidate LOY cells. We analyzed the distribution of LOY‐scores across distinct cell types and evaluated the relationship between cell proportions and LOY intensity. Furthermore, linear regression was utilized to examine the correlation between monocyte counts and the degree of LOY.

#### 2.14.4 Single‐Cell Validation of Key Protein Expression

To elucidate the cellular roles of the previously identified LOY‐associated plasma proteins, their corresponding encoding genes were mapped onto the single‐cell expression matrix. Differential expression analysis was performed between the AMD and control groups specifically within monocyte clusters. By integrating these differential signatures with cell‐type specificity metrics, we prioritized candidate molecules (e.g., IFI30 and CKB) likely mediating the biological axis between LOY and AMD through monocyte‐driven pathways.

#### 2.14.5 Local Tissue Analysis in the Choroid

To investigate the localized effects of LOY within the ocular microenvironment, we extended our analysis to a male choroidal scRNA‐seq dataset (AMD: *n* = 5; Control: *n* = 6) (Voigt et al. 2022). Across all defined cell types, we specifically examined the frequency of LOY and subsequent proportional fluctuations in resident macrophages. This analysis aimed to verify the consistency of the monocyte/macrophage system's vulnerability to LOY across both systemic circulation and local ophthalmic tissues.

### 2.15 Statistical Analysis

#### 2.15.1 Descriptive Statistics

Baseline characteristics were presented as frequencies (percentages) for categorical variables and means±SD for continuous variables. Intergroup differences across aging phenotypes or LOY status were evaluated using the Chi‐square test for categorical data, one‐way analysis of variance (ANOVA) for continuous variables across multiple groups, and independent samples *t*‐tests for two‐group comparisons.

#### 2.15.2 Logistic Regression Analysis

To examine the cross‐sectional association between LOY surrogate markers and the prevalence of five ophthalmic conditions at baseline, multivariable logistic regression models were employed. LOY status (binary), mLRRY (continuous), and LOY cell percentage (continuous) served as independent variables to estimate odds ratios (ORs) and 95% CIs.

#### 2.15.3 Linear Regression Models

Multivariable linear regression was utilized to quantify the associations between: (1) plasma protein levels and LOY surrogate markers; (2) hematopoietic indices and LOY markers; (3) monocyte counts and single‐cell LOY‐scores; and (4) LOY markers/key proteins and OCT‐derived retinal morphometric parameters.

#### 2.15.4 Cox Proportional Hazards Regression

The longitudinal impact of LOY markers on the incidence of ophthalmic diseases, as well as the association between prioritized proteins and future disease risk, was assessed using Cox proportional hazards models. Hazard ratios (HRs) and 95% CIs were estimated. To ensure the validity of the models, the proportional hazards assumption for each Cox regression was rigorously tested using Schoenfeld residuals across all exposure metrics (including mLRRY, LOY cell fraction, and binary LOY status) for each outcome disease, with the detailed evaluation results presented in Table S5.

#### 2.15.5 Restricted Cubic Spline (RCS) Analysis

To explore potential nonlinear dose–response relationships between continuous LOY markers (mLRRY and LOY cell percentage) and the risk of ophthalmic outcomes, RCS analysis was performed. We utilized four knots placed at the 5th, 35th, 65th, and 95th percentiles of the marker distribution.

#### 2.15.6 Subgroup Analyses

To assess the consistency of the observed associations across various clinical strata, subgroup analyses were conducted according to age, AMD PRS, and myopia status (SE < −0.75 D). Specifically, age was stratified using the cohort median of 58 years, and the AMD PRS was categorized based on quintiles.

Within each subgroup, fully adjusted multivariable Cox proportional hazards models (identical to Model 3, which controlled for demographic characteristics, lifestyle factors, and baseline comorbidities) were fitted, while excluding the respective stratification variable itself to avoid collinearity. To evaluate potential effect modification across strata, interaction terms between LOY metrics and the subgroup stratification variables were incorporated into the Cox models, and Wald tests were performed to calculate interaction *p* values for assessing heterogeneity.

#### 2.15.7 Mediation Analyses

A causal mediation analysis framework was implemented to evaluate whether the impact of LOY on disease risk is mediated through alterations in peripheral blood cell counts. The indirect effect, direct effect, and proportion mediated were estimated using 1000 bootstrap iterations.

#### 2.15.8 Sensitivity Analyses

To further test the robustness of our findings, sensitivity analyses were conducted: (1) by implementing a 1:10 propensity score matching (PSM) approach, matching individuals on age to re‐evaluate the core associations; (2) by incorporating general practice (GP) clinical records from the UK Biobank to identify a more comprehensive cohort of AMD patients, with the detailed diagnostic codes and re‐analysis results presented in Table S6.

### 2.16 Role of the Funding Source

The funders had no role in study design, data collection, data analysis, data interpretation, or writing of the report.

## 3 Results

### 3.1 Baseline Characteristics

A total of 223,051 male participants with high‐quality chromosomal data were identified from the UKB cohort, of whom 8920 (4.0%) were classified as LOY carriers (Table 1). At baseline, LOY carriers exhibited significantly lower mLRRY values and a concurrently higher estimated percentage of LOY cells, consistent with the predefined quantification metrics of chromosomal mosaicism.

**TABLE 1:** Baseline characteristics of study participants according to LOY status.

Variable	Total (*N* = 223,051)	LOY (*N* = 8920)	Non‐LOY (*N* = 214,131)	*p*
mLRRY	−0.01 ± 0.06	−0.19 ± 0.13	0.00 ± 0.04	< 0.001*
LOY cell percent	2.76 ± 5.35	22.05 ± 11.16	1.96 ± 2.92	< 0.001*
Age	56.75 ± 8.20	63.36 ± 5.28	56.48 ± 8.18	< 0.001*
Townsend	−1.26 ± 3.15	−1.25 ± 3.16	−1.26 ± 3.15	0.6378
BMI	27.82 ± 4.24	27.55 ± 3.96	27.84 ± 4.25	< 0.001*
PRS (AMD)	0.13 ± 1.03	0.16 ± 1.02	0.13 ± 1.03	0.013*
Ethnicity				
White	209,607 (94.0%)	8609 (96.5%)	200,998 (93.9%)	< 0.001*
Others	13,206 (5.9%)	305 (3.4%)	12,901 (6.0%)	
Education level				
Low	147,672 (66.2%)	6511 (73.0%)	141,161 (65.9%)	< 0.001*
High	75,379 (33.8%)	2409 (27.0%)	72,970 (34.1%)	
Smoking status				
Never	108,767 (48.8%)	2778 (31.1%)	105,989 (49.5%)	< 0.001*
Previous	85,473 (38.3%)	4135 (46.4%)	81,338 (38.0%)	
Current	27,700 (12.4%)	1955 (21.9%)	25,745 (12.0%)	
Alcohol intake				
Never or seldom	33,859 (15.2%)	1202 (13.5%)	32,657 (15.3%)	< 0.001*
Moderate	73,966 (33.2%)	2828 (31.7%)	71,138 (33.2%)	
Frequent	114,699 (51.4%)	4872 (54.6%)	109,827 (51.3%)	
Diabetes history				
No	206,483 (92.6%)	8163 (91.5%)	198,320 (92.6%)	< 0.001*
Yes	15,490 (6.9%)	719 (8.1%)	14,771 (6.9%)	
Hypertension history				
No	153,343 (68.7%)	5479 (61.4%)	147,864 (69.1%)	< 0.001*
Yes	69,708 (31.3%)	3441 (38.6%)	66,267 (30.9%)	
Cataract				
No	219,350 (98.3%)	8683 (97.3%)	210,667 (98.4%)	< 0.001*
Yes	3701 (1.7%)	237 (2.7%)	3464 (1.6%)	
DR				
No	222,547 (99.8%)	8905 (99.8%)	213,642 (99.8%)	0.2894
Yes	504 (0.2%)	15 (0.2%)	489 (0.2%)	
RD				
No	221,921 (99.5%)	8858 (99.3%)	213,063 (99.5%)	0.013*
Yes	1130 (0.5%)	62 (0.7%)	1068 (0.5%)	
Glaucoma				
No	222,427 (99.7%)	8882 (99.6%)	213,545 (99.7%)	0.0103*
Yes	624 (0.3%)	38 (0.4%)	586 (0.3%)	
AMD				
No	222,782 (99.9%)	8906 (99.8%)	213,876 (99.9%)	0.3932
Yes	269 (0.1%)	14 (0.2%)	255 (0.1%)	
WBC‐related items				
WBC count	6.92 ± 2.17	7.49 ± 2.16	6.90 ± 2.17	< 0.001*
Neutrophill count	4.27 ± 1.44	4.75 ± 1.59	4.25 ± 1.43	< 0.001*
Neutrophill percentage	61.19 ± 8.66	62.97 ± 8.54	61.11 ± 8.66	< 0.001*
Lymphocyte count	1.91 ± 1.30	1.92 ± 1.09	1.91 ± 1.31	0.1986
Lymphocyte percentage	27.87 ± 7.51	25.98 ± 7.28	27.95 ± 7.51	< 0.001*
Monocyte count	0.52 ± 0.23	0.57 ± 0.22	0.52 ± 0.23	< 0.001*
Monocyte percentage	7.64 ± 2.76	7.84 ± 2.81	7.63 ± 2.75	< 0.001*
Eosinophill count	0.19 ± 0.14	0.20 ± 0.15	0.19 ± 0.14	< 0.001*
Eosinophill percentage	2.76 ± 1.95	2.66 ± 1.85	2.77 ± 1.95	< 0.001*
Basophill count	0.03 ± 0.05	0.04 ± 0.05	0.03 ± 0.05	< 0.001*
Basophill percentage	0.54 ± 0.53	0.54 ± 0.56	0.54 ± 0.53	0.7359
RBC and hemoglobin				
RBC count	4.74 ± 0.38	4.64 ± 0.39	4.75 ± 0.38	< 0.001*
Hemoglobin concentration	14.99 ± 1.03	14.86 ± 1.06	14.99 ± 1.03	< 0.001*
Hematocrit percentage	43.29 ± 3.02	43.06 ± 3.17	43.30 ± 3.02	< 0.001*
Mean corpuscular volume	91.42 ± 4.49	92.95 ± 4.55	91.35 ± 4.48	< 0.001*
Mean corpuscular hemoglobin	31.66 ± 1.85	32.11 ± 1.90	31.65 ± 1.85	< 0.001*
MCHC	34.64 ± 1.06	34.54 ± 1.12	34.64 ± 1.05	< 0.001*
RBC distribution width	13.45 ± 0.89	13.50 ± 0.90	13.45 ± 0.89	< 0.001*
Reticulocyte‐related items				
Reticulocyte count	0.07 ± 0.04	0.06 ± 0.03	0.07 ± 0.04	0.0161*
Reticulocyte percentage	1.38 ± 0.88	1.39 ± 0.61	1.38 ± 0.89	0.3486
Mean reticulocyte volume	106.34 ± 7.81	107.86 ± 7.72	106.28 ± 7.81	< 0.001*
Immature reticulocyte fraction	0.29 ± 0.06	0.30 ± 0.06	0.29 ± 0.06	< 0.001*
HLS reticulocyte count	0.02 ± 0.01	0.02 ± 0.01	0.02 ± 0.01	< 0.001*
HLS reticulocyte percentage	0.41 ± 0.30	0.43 ± 0.21	0.41 ± 0.30	< 0.001*
Platelet‐related items				
Platelet count	238.01 ± 56.02	243.99 ± 59.02	237.76 ± 55.87	< 0.001*
Platelet crit	0.22 ± 0.05	0.22 ± 0.05	0.22 ± 0.04	< 0.001*
Mean platelet volume	9.29 ± 1.07	9.28 ± 1.08	9.29 ± 1.07	0.546
Platelet distribution width	16.57 ± 0.53	16.57 ± 0.52	16.57 ± 0.53	0.9813
Others				
Mean sphered cell volume	82.74 ± 5.32	84.37 ± 5.62	82.67 ± 5.30	< 0.001*
Nucleated RBC count	0.00 ± 0.03	0.00 ± 0.03	0.00 ± 0.03	0.8365
Nucleated RBC percentage	0.03 ± 0.44	0.03 ± 0.42	0.03 ± 0.44	0.5937

*Note:* Continuous variables are presented as mean ± standard deviation (SD), and categorical variables are presented as number (percentage). LOY index includes mLRRY (median log R ratio of chromosome Y) and estimated LOY cell percentage. Ophthalmic conditions include cataract, DR (diabetic retinopathy), RD (retinal detachment), glaucoma, and AMD (age‐related macular degeneration). Hematological parameters encompass WBC (white blood cell), RBC (red blood cell), reticulocyte, and platelet lineages. PRS (AMD) represents the standardized polygenic risk score for AMD. *p* Values were calculated using independent samples *t*‐tests or ANOVA for continuous variables and Chi‐square tests for categorical variables. Asterisks (*) denote statistical significance at *p* < 0.05.

Abbreviations: HLS, high light scatter; MCHC, mean corpuscular hemoglobin concentration.

Detailed comparative analysis of socio‐demographic and clinical profiles revealed that LOY carriers were characterized by more advanced age, lower BMI, a higher proportion of White ethnicity, and lower educational attainment. Furthermore, LOY status was associated with a higher prevalence of tobacco and alcohol consumption, as well as a greater burden of metabolic comorbidities, including diabetes and hypertension. Regarding ophthalmic health at baseline, LOY carriers demonstrated significantly higher prevalence rates of cataract, retinal detachment, and glaucoma. Notably, while the baseline prevalence of AMD did not differ significantly between the two groups, LOY carriers possessed a markedly higher PRS for AMD, suggesting an elevated underlying genetic susceptibility.

Besides, we evaluated peripheral blood indices across the cohort. LOY carriers exhibited significantly elevated counts of white blood cells, neutrophils, and monocytes. Conversely, erythrocyte counts and hemoglobin concentrations were slightly reduced, accompanied by a substantial increase in MCV. These systemic shifts across multiple hematopoietic lineages suggest that LOY may be intrinsically linked to a state of altered bone marrow homeostasis and dysregulated myeloid differentiation.

### 3.2 Association of LOY With Age‐Related Ophthalmic Conditions

To elucidate the impact of LOY on ophthalmic health, we performed systematic evaluations across both cross‐sectional and longitudinal frameworks. In the cross‐sectional analysis, all three LOY indices—comprising mLRRY, estimated LOY cell percentage, and binary LOY status—exhibited robust positive associations with the prevalence of all five ophthalmic diseases in unadjusted models (Table S3). However, these associations were largely attenuated upon full adjustment for covariates, suggesting that the initial correlations were primarily driven by age‐related confounding rather than an independent cross‐sectional effect.

In contrast, longitudinal assessments revealed a distinct and specific predictive role for LOY. Survival analysis among the PSM‐matched patients (standardized mean difference = 0.0799; detailed in Table S4) revealed that LOY carriers demonstrated significantly elevated risks for incident AMD (*p* = 0.009) and cataract (*p* = 0.0017; Figure 1A–E). Notably, following stringent adjustment for all socio‐demographic and clinical covariates, a higher continuous mLRRY was uniquely and significantly associated with a reduced risk of incident AMD (HR: 0.611, 95% CI: 0.397–0.942; Table 2), whereas binary LOY status showed a marginal association with incident AMD in the fully adjusted model (HR: 1.124, 95% CI: 0.995–1.269, *p* = 0.060; Table 2). The proportional hazards assumption for these models was successfully verified using Schoenfeld residuals (Table S5). This association remained robust among AMD patients after further taking GP diagnoses into consideration (Table S6). Other ophthalmic conditions did not maintain statistical significance in the fully adjusted Cox models (Table S7), while consistent trends were observed among the PSM cohort (Table S8), collectively demonstrating the robustness of these findings and highlighting the specific vulnerability of the retinal microenvironment to Y‐chromosome mosaicism.

**FIGURE 1:**
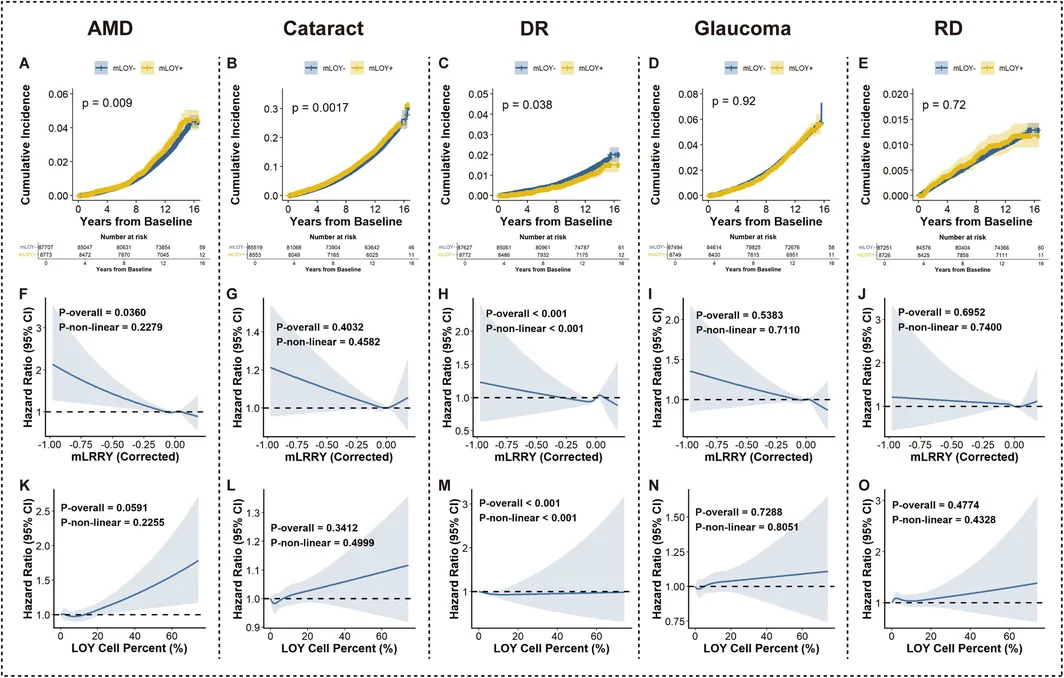
Longitudinal association and dose–response relationship between mLOY and ophthalmic conditions. Forest plots in panels (A–E) illustrate the longitudinal association between LOY status and the risk of five incident ophthalmic conditions, for which a 1:10 propensity score matching (PSM) protocol was implemented to balance baseline age between groups (resulting in a mean age of 63.4 years in the LOY group versus 62.9 years in the non‐LOY group). Meanwhile, panels (F–J) and (K–O) present restricted cubic spline (RCS) curves depicting the relationship between continuous mLRRY values or estimated LOY cell percentages and the hazard of these diseases, respectively. All statistical models were adjusted for a comprehensive set of socio‐demographic factors and clinical comorbidities to account for potential confounding.

**TABLE 2:** Longitudinal association between mLOY indices and the risk of incident AMD.

LOY index	Model	N_Total	N_Incident	HR	LCI	UCI	*p*
mLRRY	Crude	222,782	3856	0.0605	0.0427	0.0858	< 0.001*
mLRRY	Model1	221,317	3837	0.5891	0.3835	0.9048	0.0156*
mLRRY	Model2	220,259	3807	0.6521	0.4226	1.0064	0.0534
mLRRY	Model3	219,531	3796	0.6114	0.3969	0.9420	0.0257*
LOY status	Crude	222,782	3856	2.1596	1.9175	2.4324	< 0.001*
LOY status	Model1	221,317	3837	1.1339	1.0052	1.2790	0.0409*
LOY status	Model2	220,259	3807	1.1101	0.9833	1.2532	0.0914
LOY status	Model3	219,531	3796	1.1235	0.9950	1.2687	0.0602
LOY cell percent	Crude	222,782	3856	1.0316	1.0276	1.0356	< 0.001*
LOY cell percent	Model1	221,317	3837	1.0051	1.0006	1.0097	0.0259*
LOY cell percent	Model2	220,259	3807	1.0040	0.9994	1.0086	0.0894
LOY cell percent	Model3	219,531	3796	1.0046	1.0001	1.0092	0.0461*

*Note:* Cox proportional hazards regression models were used to estimate hazard ratios (HRs) and 95% confidence intervals (CIs). mLRRY: median log R ratio of chromosome Y; LOY status: binary classification (Carrier vs. Non‐carrier); LOY cell percent: estimated cellular mosaicism fraction. N_Total: total number of participants included in each model; N_Prevalent: number of incident AMD cases during the follow‐up period. Crude: unadjusted model. Model 1: adjusted for age, quadratic term of age, ethnicity, Townsend Deprivation Index, education level, and BMI. Model 2: adjusted for variables in Model 1 plus smoking status, and alcohol intake. Model 3: adjusted for variables in Model 2 plus history of hypertension and diabetes mellitus. Values in bold and marked with an asterisk (*) indicate statistical significance (*p* < 0.05).

Abbreviations: LCI, lower limit of the 95% CI; UCI, upper limit of the 95% CI.

To delineate the precise dose–response relationship, we implemented RCS analysis, which revealed a significant nonlinear inverse association between mLRRY and incident AMD (*p* for overall = 0.0245; Figure 1F). While other ophthalmic conditions exhibited comparable downward trends, none achieved statistical significance as their 95% CIs remained consistent with the null hypothesis (Figure 1G–J). Specifically, for mLRRY, a critical risk threshold was identified at −0.17, beyond which the hazard for AMD escalated significantly. Correspondingly, the estimated LOY cell percentage exhibited a significant positive correlation with AMD risk, with a marked increase in hazard observed once the mosaicism fraction surpassed 25.5% (Figure 1K–O). These data collectively characterize LOY as a robust, independent longitudinal determinant uniquely associated with AMD pathogenesis.

### 3.3 Subgroup Analyses by Myopia, Genetic Liability, and Age

To evaluate the consistency of the association between LOY and incident AMD across diverse clinical strata, we performed systematic subgroup analyses categorized by refractive status (myopia defined as SE < −0.75 D), PRS quintiles, and age groups (Figure 2 and Table S9). Our findings identified significant effect modification, particularly by ocular physiology and age. The inverse association between mLRRY and AMD risk was notably more pronounced among non‐myopic individuals (HR: 0.919; 95% CI: 0.867–0.973, FDR‐corrected *p* = 0.0149) and participants in the older age stratum (HR: 0.934; 95% CI: 0.911–0.957, FDR‐corrected *p* = 1.49 × 10^−6^).

**FIGURE 2:**
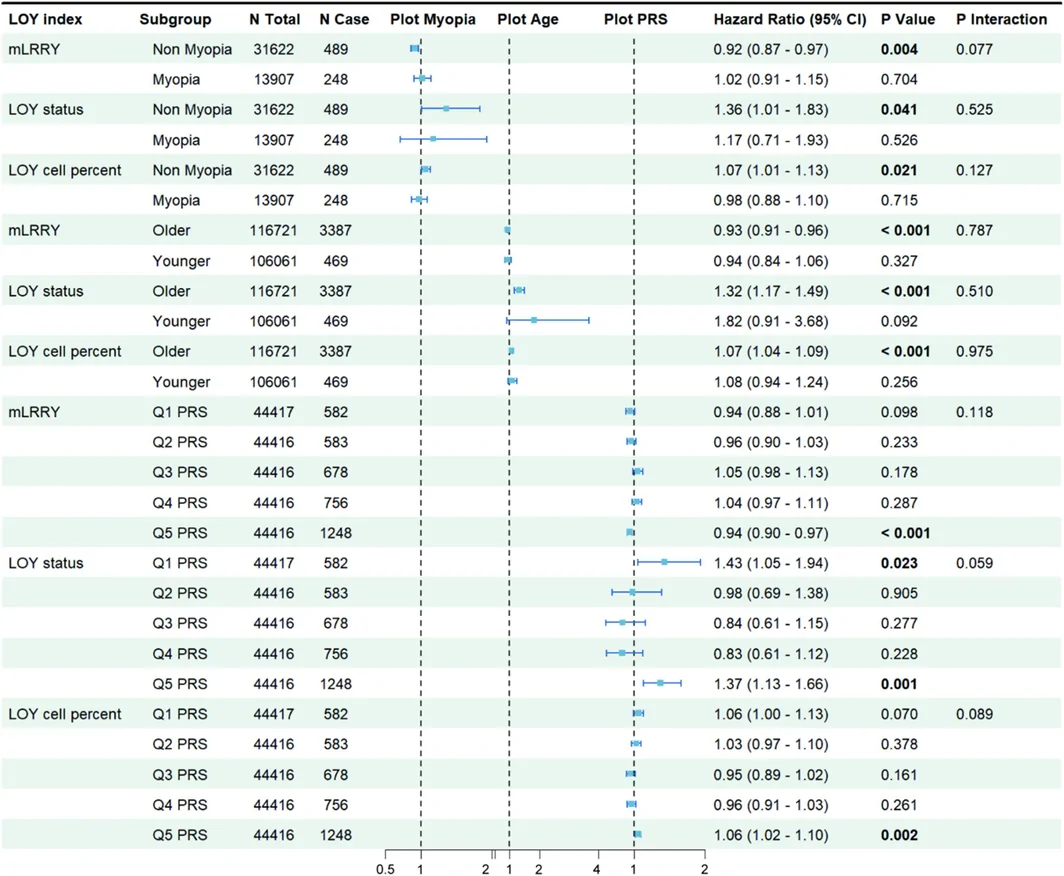
Subgroup analyses of the association between mLRRY and incident AMD. This forest plot displays the association between mLRRY and the risk of incident AMD across various clinical strata. Participants were stratified by age (using the cohort median of 58 years), refractive status (myopia defined as spherical equivalent [SE] < −0.75 D), and quintiles of polygenic risk scores (AMD PRS). Within each subgroup, fully adjusted multivariable Cox models (identical to Model 3) were fitted while excluding the respective stratification variable to avoid collinearity. *p* values for interaction were calculated using Wald tests to evaluate potential effect modification by these clinical characteristics.

Furthermore, we examined the interplay between stochastic chromosomal loss and germline genetic predisposition. Although correlation analyses revealed negligible associations between the AMD PRS and LOY metrics (e.g., *r* = −0.005 for mLRRY and *r* = 0.004 for LOY cell fraction, with uniform distributions across PRS quintiles; Figure S1A–D), the HR of LOY varied across genetic strata. Notably, the association of mLRRY with AMD was most prominent among individuals in the highest PRS quintile (Q5) (HR: 0.938; 95% CI: 0.903–0.974; *p* = 0.0046). Although formal interaction testing across PRS strata did not reach a traditional significance threshold (*p* for interaction = 0.118), the concentration of risk‐associated signals within the high‐genetic‐liability group suggests that LOY may act as a critical clinical indicator in genetically vulnerable populations.

### 3.4 Proteomic Signatures Interconnecting LOY

To identify the most robust proteomic signatures associated with LOY, we performed a comparative analysis across three distinct LOY definitions: mLRRY, estimated LOY cell percentage, and binary LOY status. Following FDR correction, 137 proteins exhibited significant associations across all three metrics (FDR‐corrected *p* < 0.05; Tables S10–S12 and Figure S2A–C). This shared proteomic profile featured critical mediators of inflammatory response and myeloid activity, including GDF15, MMP12, MPO, and S100A12. The high degree of cross‐metric consistency underscores these factors as core candidate targets for elucidating the downstream biological mechanisms of LOY. Additionally, 135 and 73 proteins showed significant correlations with two and one LOY indices, respectively (Figure S2E).

To further investigate the longitudinal association between circulating proteins and AMD risk, we conducted Cox proportional hazards regression on 2920 plasma proteins. Utilizing a dual‐stringency criterion (FDR‐corrected *p* < 0.05 and log_2_HR > 0.1), we prioritized 393 proteins significantly associated with incident AMD (Table S13 and Figure S2D). Among these, VSIG4, CFHR5, GDF15, and TIMP4 emerged as primary risk‐promoting factors, while CBLN4, NTF3, LRRN1, and CNTN3 were associated with reduced risk. Functional enrichment analysis revealed that these robustly associated proteins were highly concentrated in pathways governing complement and immunomodulation (e.g., CFHR4/5 and CD46/55), inflammation and myeloid response (e.g., CCL3/4 and CD163), and extracellular matrix remodeling (e.g., MMP12 and TIMP1/4). These findings validate the pivotal roles of complement overactivation and systemic inflammation in AMD pathogenesis and provide a molecular foundation for how LOY may drive ocular disease through the dysregulation of leukocyte function.

By intersecting 345 proteins associated with at least one LOY index with those linked to incident AMD, we identified 65 candidate protein mediators (Table S14). Assuming a directional causality from LOY to AMD, we further narrowed our focus to 55 proteins exhibiting concordant effect directions, comprising 15 protective factors and 40 risk‐associated factors. Cross‐validation across all three LOY metrics identified a highly robust subset of 23 core proteins. On one hand, inflammatory stress markers (e.g., GDF15 and LCN2) and ECM‐degradative enzymes (e.g., MMP12 and PLAUR) significantly elevated AMD risk, suggesting that LOY may drive disease progression via the induction of chronic systemic inflammation. On the other hand, factors such as ATRN, CNTN1, and BCAN demonstrated consistent protective effects, potentially by maintaining neurological and tissue homeostasis. This hierarchical consistency not only pinpoints critical risk determinants but also provides compelling molecular evidence for a model in which LOY triggers AMD by modulating complement activation and the immune microenvironment.

### 3.5 Temporal Evolution and Trajectory Clustering of LOY‐Associated Proteins

We further employed LOESS regression to characterize the temporal trajectories of LOY‐associated plasma proteins leading up to the clinical diagnosis of AMD. Our analysis identified 41 prioritized proteins that exhibited significant quantitative deviations during the preclinical phase. The temporal sequence of these alterations, defined by an absolute *Z*‐score exceeding 0.25, was mapped over a 15‐year window prior to diagnosis (Table S15). We observed that CHGA maintained sustained pathological elevation throughout the prodromal period, whereas CEACAM16 and WFIKKN1 displayed early‐onset depletion. Notably, MMP12 concentrations deviated significantly approximately 12 years before diagnosis, exhibiting a progressive and accelerating upward trajectory that persisted until the clinical endpoint (Figure 3A).

**FIGURE 3:**
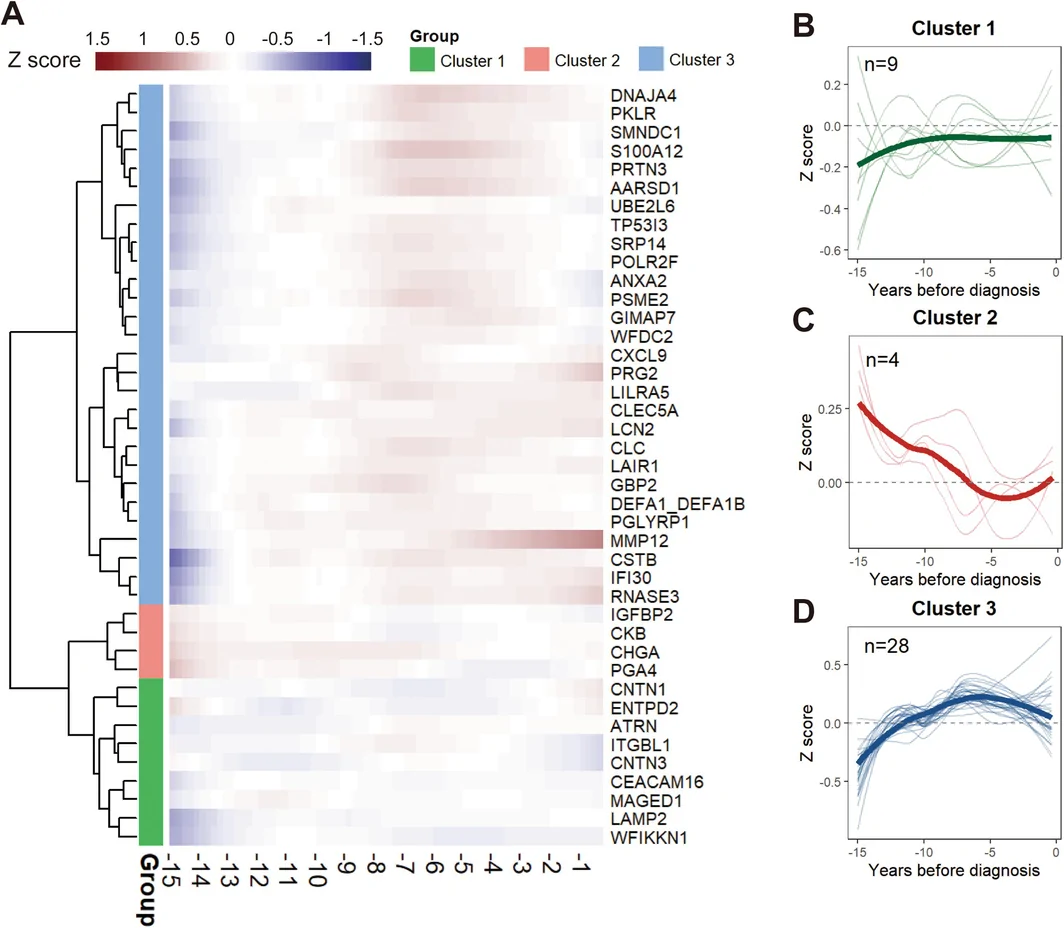
Temporal trajectories and clustering of LOY‐associated plasma proteins. Panel (A) presents a sequential timeline of prioritized plasma proteins that reached a defined threshold of clinical abnormality during the 15‐year window preceding AMD diagnosis, while panels (B–D) show the results of unsupervised hierarchical clustering used to group these proteins based on their temporal expression profiles. All trajectories were modeled using locally estimated scatterplot smoothing (LOESS) regression to capture nonlinear kinetic patterns.

To reduce proteomic complexity and identify shared pathological patterns, we applied unsupervised hierarchical clustering, which partitioned the 41 proteins into three distinct trajectory clusters consisting of 9, 4, and 28 proteins, respectively (Figure 3B–D). These clusters predominantly manifested nonlinear kinetic profiles. Cluster 1, which included CEACAM16 and WFIKKN1, was characterized by a general trend of early protein depletion. Cluster 2 exhibited a U‐shaped trajectory, with levels initially declining before rising closer to the time of diagnosis. Conversely, Cluster 3 followed an inverted U‐shaped pattern, with protein levels increasing during the mid‐preclinical phase and reaching peak *Z*‐scores approximately 6 years prior to the clinical manifestation of AMD.

### 3.6 Functional Enrichment of LOY‐Associated Proteomes

To elucidate the biological properties of the prioritized proteins, we performed GO enrichment analyses (Table S16). Within the biological process domain, these proteins were significantly enriched in defense responses to bacteria and antimicrobial humoral immunity, suggesting that systemic immune dysregulation and chronic inflammation are pivotal in the LOY‐driven pathogenesis of AMD. Cellular component analysis revealed a high concentration of these proteins in secretory granule lumens—specifically azurophil, specific, and ficolin‐1 granules—as well as the collagen‐containing extracellular matrix. These findings imply that immune cell degranulation may serve as a critical mechanistic link in disease progression. In terms of molecular function, the proteins were primarily involved in endopeptidase and peptidase regulator/inhibitor activities, particularly those of serine‐ and cysteine‐type endopeptidases. This further confirms that a disrupted proteolytic balance is closely associated with tissue remodeling in AMD.

### 3.7 Hematopoietic Mediation Between LOY and Ophthalmic Disease

Given the strong implication of immune cell involvement, and consistent with literature suggesting LOY exerts pathogenic effects through myeloid cells (Sano et al. 2022), we evaluated the correlation between LOY indices and peripheral blood cell lineages. Linear regression models revealed that mLRRY exerts a broad and significant impact on the hematopoietic profile, characterized by a substantial decline in myeloid cell abundance (Table S17). Notably, mLRRY exhibited a marked inverse correlation with both neutrophil counts (*β* = −1.204) and total white blood cell counts (*β* = −1.336), alongside a significant reduction in platelet counts (*β* = −37.52) as mLRRY increased. Regarding the erythroid system, while erythrocyte counts showed a marginal increase, there was a significant reduction in MCV and suppression of IRF, indicating a pronounced developmental shift in hematopoietic precursors under the LOY background. Consistent with prior evidence, the association between mLRRY and monocyte counts was highly robust (*β* = −0.115, adjusted *p* = 4.70 × 10^−42^), validating the monocyte lineage as a key target of LOY.

Causal mediation analysis further quantified the degree to which various hematopoietic components bridge the association between mLRRY and incident AMD (Table S18). Monocyte counts exhibited a significant mediating effect, accounting for approximately 2.53% of the total effect (Figure 4A). Within the leukocyte sub‐lineages, neutrophils and lymphocyte percentages demonstrated more prominent contributions, with mediation proportions of 5.39% and 5.68%, respectively (Figure 4B,C). Additionally, erythrocyte‐related indices contributed approximately 5.58% to the observed mediation effect (Figure 4D).

**FIGURE 4:**
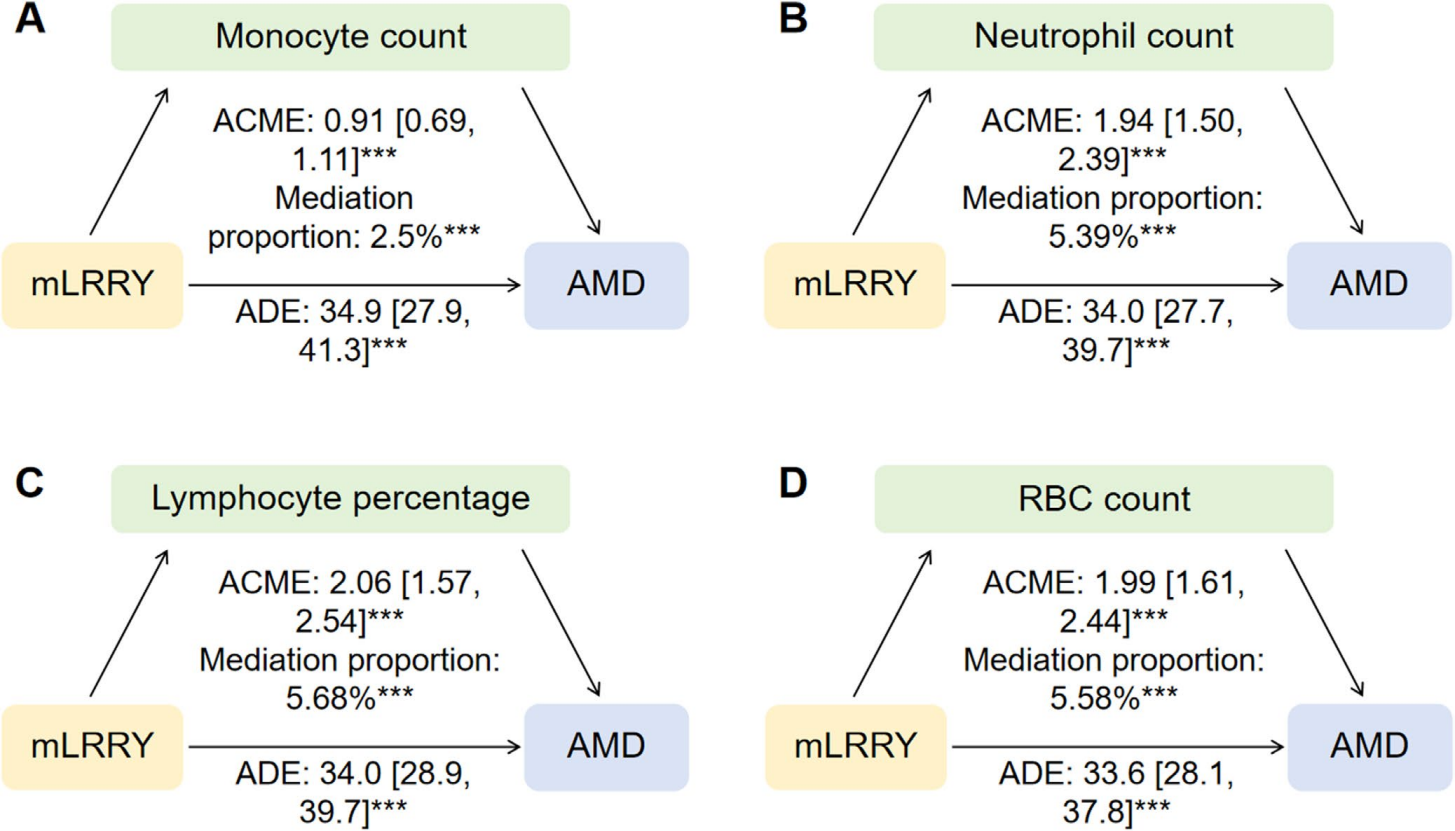
Mediation of LOY‐driven AMD risk via hematopoietic lineages. These path diagrams and bar charts illustrate the causal mediation analysis framework used to evaluate the potential mediating roles of monocyte counts (A), neutrophil counts (B), lymphocyte percentage (C), and erythrocyte‐related indices (D) on the association between mLRRY and incident AMD. Effect estimates and their corresponding 95% confidence intervals were derived through the implementation of bootstrap iterations.

To further validate the causal role of monocyte traits suggested by our mediation analysis, we performed a two‐sample MR analysis. MR analysis showed that specific monocyte immunophenotypes and counts are genetically associated with the risk of AMD (Table S19). In the Finn‐AMD cohort, the IVW method showed that the surface marker CD14 on CD33^br^HLA^−^DR^+^CD14^dim^ was significantly and positively correlated with AMD risk (*p* < 0.05; Figure S3). In the MVP‐AMD cohort, the absolute count of HLA‐DR^++^ monocytes was significantly and positively correlated with AMD risk (*p* < 0.05; Figure S3). The weighted median method and other complementary MR methods showed consistent effect directions for some core phenotypes (Figure S4).

### 3.8 Single‐Cell Transcriptomic Validation in Human AMD Cohorts

To validate the mechanistic link between LOY and AMD pathogenesis, we integrated and analyzed scRNA‐seq data from the peripheral blood of 18 male patients with AMD and 9 matched male controls. Given prior evidence implicating monocytes and macrophages as primary drivers of AMD (Xue et al. 2021; Daftarian et al. 2020) and potential mediators of LOY‐related pathologies (Voigt et al. 2022), we hypothesized these myeloid populations to be the critical effector cells. Using canonical marker genes, we annotated 11 distinct cell lineages (Figures 5A and S5A,B, and Table S20), focusing on CD14+ monocytes (LYZ and CD14) and CD16+ monocytes (MS4A7, FCGR3A, and CD68).

**FIGURE 5:**
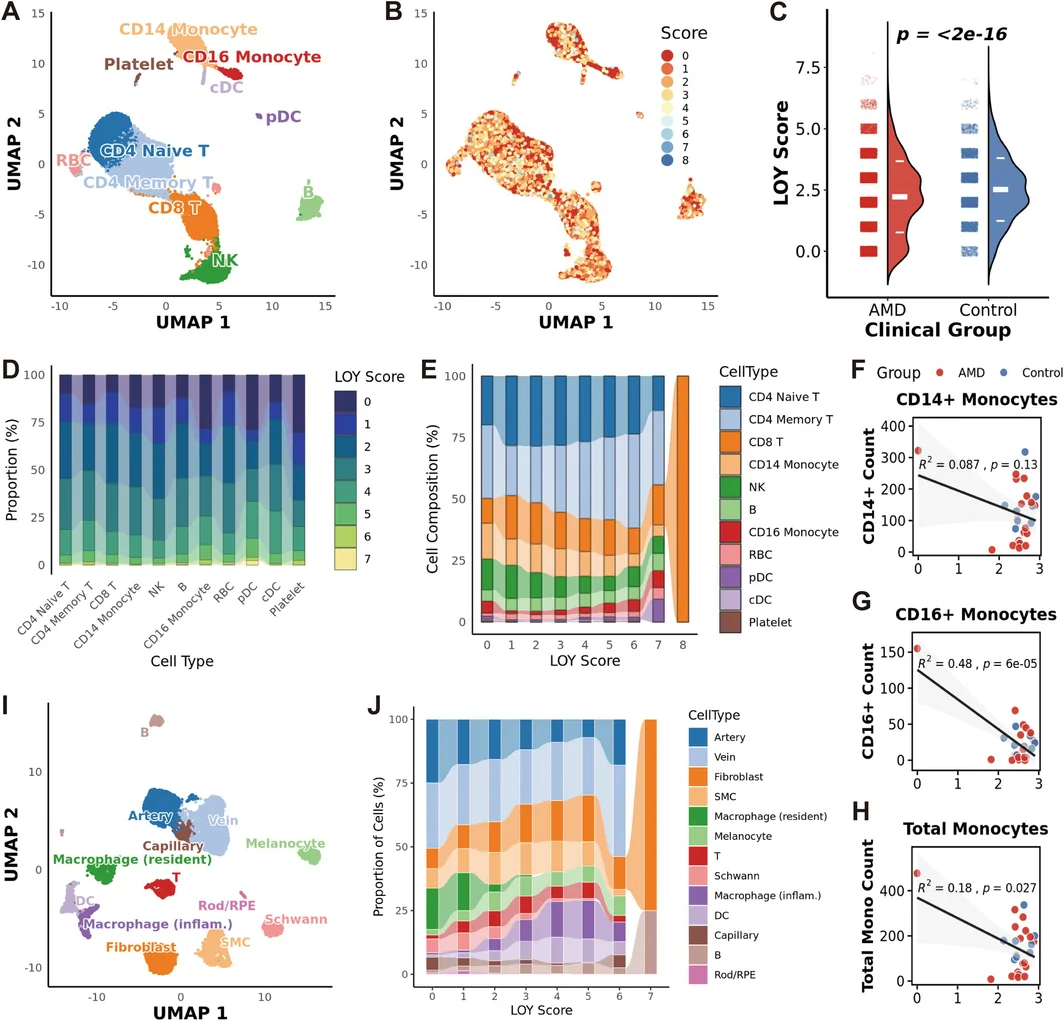
Single‐cell transcriptomic validation of mLOY in peripheral blood and choroid. (A) UMAP visualization of integrated peripheral blood mononuclear cells (PBMCs) from AMD patients (*n* = 18) and controls (*n* = 9), annotated into 11 cell lineages. (B) UMAP plot colored by individual cell LOY‐scores, calculated based on the cumulative expression of 10 Y‐chromosome‐specific genes. (C) Violin and box plots comparing the distribution of single‐cell LOY‐scores between the AMD and control groups. (D) Bar chart showing the distribution of LOY‐scores across identified cell types. (E) Stacked bar plot illustrating the cell composition of various PBMC lineages as a function of the LOY‐score. (F–H) Scatter plots and linear regression analyses depicting the correlation between monocyte counts (CD14+, CD16+, and Total) and LOY‐scores. (I) UMAP visualization of human choroidal tissues annotated into 13 distinct cell types. (J) Proportional distribution of choroidal cell types across different LOY‐score levels, highlighting the dynamics of resident macrophages.

Individual cell LOY‐scores were quantified based on the expression of Y‐chromosome‐specific genes (Figure 5B), revealing an inverted U‐shaped relationship with cell proportions (Figure S5C). AMD patients exhibited significantly lower mean LOY‐scores and a markedly higher frequency of cells with absolute Y‐loss compared to controls (Figures 5C and S5D,E). This observation appeared to be independent of age, as we found no significant linear correlation between age and individual cellular LOY‐weighted scores (Figure S6A). Among the identified lineages, CD16+ monocytes and platelets demonstrated the highest prevalence of complete Y‐loss (Figure 5D). Notably, the proportion of monocytes was inversely correlated with increasing LOY‐scores (Figure 5E). Scatter plot analysis further confirmed a significant negative correlation between monocyte counts and LOY‐scores (Figure 5F–H), particularly within the CD16^+^ subset (*R* ^2^ = 0.48, *p* = 6 × 10^−5^). Furthermore, Figure S6B–D revealed a significant negative correlation between CD16^+^ LOY scores and age (*R* ^2^ = 0.177, *p* = 0.036), reinforcing our population‐level findings from the UKB and identifying monocytes, especially CD16^+^ monocytes, as primary cellular targets of LOY.

We further cross‐referenced the 41 previously identified LOY‐associated plasma proteins within the monocyte transcriptomes. Twenty‐nine of these candidates were detected at the single‐cell level. Monocytes from AMD patients displayed significant upregulation of GBP2, AARSD1, ANXA2, CSTB, GIMAP7, IFI30, LAIR1, POLR2F, PSME2, UBE2L6, CKB, and IGFBP2, while WFIKKN1 was significantly downregulated (Figure S7). Notably, IFI30 and CKB exhibited monocyte‐specific expression profiles (Figure S8), suggesting their potential role as key molecular checkpoints in the monocyte‐mediated biological axis linking LOY to AMD.

To determine whether the effects of LOY extend beyond systemic circulation, we additionally conducted a parallel scRNA‐seq analysis of choroidal tissues from male AMD patients and controls. Within the 13 annotated choroidal cell types (Figure 5I and Table S21), resident macrophages exhibited the highest frequency of LOY (Figure S5F). Furthermore, a progressive decrease in resident macrophage proportions was observed in relation to increasing LOY‐scores (Figure 5J). These localized findings parallel the systemic shifts observed in peripheral blood, demonstrating that the monocyte/macrophage system is susceptible to the impact of LOY both in circulation and within the local ophthalmic microenvironment.

### 3.9 Structural Correlation Between LOY Indices, Candidate Proteins, and Retinal Microarchitecture

Following the confirmation of LOY's impact on the systemic and local monocyte/macrophage axis, we conducted a comprehensive heatmap‐based correlation analysis to elucidate the associations between the 41 prioritized proteins, LOY surrogate markers, and 40 retinal morphometric parameters derived from OCT. The results revealed extensive and statistically significant inverse correlations between multiple core proteins—exemplified by LILRA5 and CLEC5A—and the thickness of various retinal layers (Table S22). These negative associations were particularly pronounced in macular thickness and critical structural traits, including the interval from the INL to the RPE (Figure S9). These findings suggest that the elevation of such pro‐inflammatory and immune‐related proteins may directly contribute to the pathological process of retinal atrophy.

Concurrently, LOY surrogate indices demonstrated a consistent inverse relationship with retinal thickness, achieving statistical significance across multiple topographic subfields (including the RNFL and RPE thickness; Figure S9 and Table S23). This structural convergence reinforces the hypothesis that LOY‐associated plasma proteins are intrinsically linked to specific retinal regions involved in AMD pathogenesis, underscoring a complex interplay between mosaic chromosomal loss and ocular degenerative phenotypes.

### 3.10 Predictive Performance of Prioritized Proteomic Signatures for Future Risk of Ophthalmic Disease

To identify a parsimonious yet high‐performing biomarker panel, we executed a feature importance ranking based on the LightGBM gain mechanism, integrated with a PFS strategy across the 41 candidate proteins. During the feature selection phase, model performance exhibited a steady incremental improvement as proteins were iteratively incorporated. The cumulative AUC in a 5‐fold cross‐validation peaked upon the inclusion of the top 14 key proteins, headlined by LAIR1, thereby defining the optimal feature subset (Figure 6A). This rigorous selection process effectively balanced model complexity with predictive power, establishing a robust foundation for longitudinal risk assessment.

**FIGURE 6:**
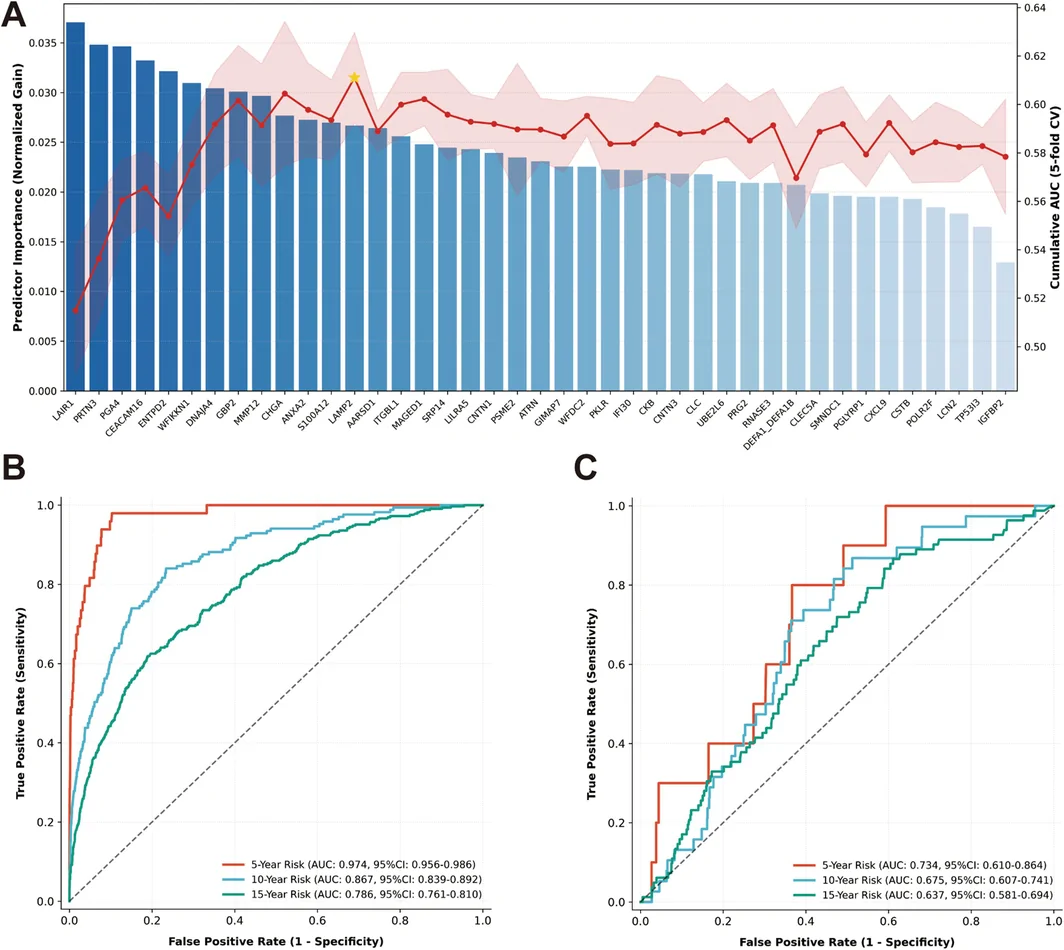
Machine learning framework for Olink proteomic risk prediction of AMD. (A) Feature selection process displaying candidate proteins ranked by LightGBM gain‐based importance (bars) and the cumulative AUC of the prognostic model during 5‐fold cross‐validation (line) using a progressive forward selection (PFS) strategy. (B) Receiver operating characteristic (ROC) curves of the finalized proteomic model in the training cohort for predicting AMD risk at 5, 10, and 15 years. (C) ROC curves demonstrating the predictive performance and discriminative capacity of the model in the independent validation cohort across multiple temporal scales.

The resulting prognostic model, constructed using the optimal protein panel, demonstrated superior discriminative capacity across multiple temporal scales. In the training cohort, the model achieved AUCs of 0.974 (95% CI: 0.956–0.986), 0.867 (95% CI: 0.839–0.892), and 0.786 (95% CI: 0.761–0.810) for predicting AMD risk at 5, 10, and 15 years, respectively (Figure 6B). Subsequent validation in an independent test set yielded a 5‐, 10‐, and 15‐year incident risk AUC of 0.734 (95% CI: 0.610–0.864), 0.675 (95% CI: 0.607–0.741), and 0.637 (95% CI: 0.581–0.694), respectively (Figure 6C). These findings confirm the model's reliable generalization performance and underscore its predictive stability across extended time horizons, suggesting significant potential for the early identification of individuals at high risk for AMD.

We additionally investigated whether incorporating mLRRY provided incremental predictive value for incident AMD over and above LOY‐associated proteins or AMD‐PRS. In the independent validation set, the time‐dependent AUCs for predicting 5‐, 10‐, and 15‐year AMD risk using the mLRRY single metric alone were 0.547, 0.536, and 0.488, respectively (Figure S10). Specifically, incorporating mLRRY into the LOY‐associated protein models yielded comparable or slightly enhanced predictive discrimination across time horizons (e.g., 5‐year train AUC: 0.978 vs. 0.974; Figure S11), whereas adding mLRRY to the AMD‐PRS models provided noticeable incremental utility for AMD incidence prediction (e.g., 5‐year train AUC: 0.837 vs. 0.789; Figure S12). These findings suggest that the predictive performance of mLRRY is relatively limited when used alone for individual risk prediction, whereas its combination with other predictors can enhance predictive utility.

## 4 Discussion

In this study, we integrated a large‐scale prospective cohort with multi‐omic analyses to systematically elucidate the association between LOY and age‐related ophthalmic diseases. First, we established a robust longitudinal association between peripheral blood LOY and incident AMD risk, which remained consistent across genetic and refractive strata. Second, by merging plasma proteomic trajectories with single‐cell transcriptomics, we identified monocyte‐related systemic inflammation as the critical nexus linking somatic mosaicism to retinal microenvironment alterations. Furthermore, the correlation between LOY‐related proteins and retinal morphometry validated its impact on subclinical structural degeneration. Finally, a protein‐based prognostic model demonstrated significant clinical utility in predicting disease onset 5 years in advance. Collectively, this research provides a comprehensive framework for understanding how somatic mutations drive sensory degradation, offering transformative strategies for the precision management of age‐related ophthalmic diseases.

Previous studies have established a robust association between peripheral blood LOY and various cancers (Abdel‐Hafiz et al. 2023; Thompson et al. 2019), respiratory diseases (Weng et al. 2024), and cardiovascular diseases (Sano et al. 2022; Hussain et al. 2026). While cross‐sectional evidence has previously suggested a link between LOY and AMD (Grassmann et al. 2019; Duan et al. 2022), our study leverages the large‐scale prospective UK Biobank cohort to significantly strengthen the evidence for a longitudinal association, identifying LOY as an independent risk factor of incident AMD. We observed that AMD risk increases significantly when mLRRY falls below −0.17 or the LOY mosaicism exceeds 25.5%. It indicated that once hematopoietic mutant clones reach a specific threshold, they may compromise the homeostatic compensation of the retinal microenvironment (Liisborg 2022), thereby facilitating the transition from physiological aging to pathological degeneration. Furthermore, while initial survival analyses associated LOY with multiple aging‐related ocular conditions, subsequent Cox and RCS models confirmed significance exclusively for AMD. This divergence potentially reflects a more direct pathophysiological link between AMD and LOY‐induced systemic immune dysregulation (Khan et al. 2023; Lin et al. 2024; Voigt et al. 2022; Gunay 2024), whereas other ophthalmic disorders may be predominantly driven by localized anatomical or non‐immunological factors.

Consistent with the observations by Sano et al. (2022) and Chen, Shen, et al. (2025) in the cardiovascular system, our findings suggest that LOY is not merely a passive marker of biological aging but may actively contribute to organ damage by modulating systemic pathophysiological processes, particularly through circulating myeloid cells. Population‐level analysis of hematological indices revealed a pronounced myeloid bias in LOY carriers, characterized by significant elevations in monocyte and neutrophil counts. This systemic immune shift provided a logical framework for transitioning from epidemiological associations to cellular mechanisms. Through scRNA‐seq, we observed the highest frequency of LOY within monocytes and choroidal resident macrophages, accompanied by the differential up‐regulation of genes such as IFI30 and CKB. IFI30 (lysosomal thiol reductase) plays a pivotal role in antigen processing and inflammatory signaling (Zhang et al. 2024; Ewanchuk and Yates 2018), while CKB (creatine kinase B‐type) is closely linked to cellular energy metabolism (He et al. 2024; Rahbani et al. 2021) and macrophage activation (Murata et al. 2017). This evidence chain supports a potential pathological axis wherein Y‐deficient myeloid cells acquire a pro‐inflammatory phenotype; these cells may secrete inflammatory mediators or infiltrate the choroid via the systemic circulation, subsequently triggering immune response and tissue remodeling. Our mediation analysis further quantifies the contribution of monocytes to this process, suggesting a biological pathway whereby somatic mutations in the hematopoietic system correlate with the progression of retinal pathology through a myeloid‐mediated axis.

The 41 LOY‐associated proteins identified in this study exhibit a highly consistent pathological logic. Functional enrichment analysis revealed that these proteins are significantly involved in defense responses, secretory granule lumens, and the regulation of endopeptidase activities. These findings suggest that immune cell degranulation and the resulting proteolytic imbalance are pivotal mechanisms driving tissue remodeling in AMD (Obasanmi et al. 2024; Dabouz et al. 2024; Mcharg et al. 2022). Through LOESS trajectory modeling, we found that MMP12 concentrations deviated significantly approximately 12 years prior to clinical diagnosis. As a protease capable of degrading elastin and remodeling the underlying structure of the RPE, its sustained pathological exposure likely leads to the progressive attrition of the Bruch's membrane and retinal barrier function (Yi et al. 2022; Wang, Zhou, et al. 2025). Furthermore, the expression fluctuations of LAIR1, an inhibitory immune receptor and the top‐ranking feature in our predictive model, directly reflect the disruption of systemic immune homeostasis (Pascoal Ramos et al. 2025). CHGA (Chromogranin A) maintained sustained pathological elevation throughout the prodromal period, reflecting chronic impairment of neuroendocrine and stress responses (Sun, Chen, et al. 2023); conversely, the early‐onset depletion of WFIKKN1 may compromise its capacity to inhibit specific proteolytic activities (Monestier and Blanquet 2016). This temporal logic underscores that systemic proteomic dysregulation serves as an early initiating event in AMD pathogenesis rather than a secondary phenomenon. A machine learning model constructed using 14 key proteins demonstrated robust predictive performance over a 5‐year horizon, offering a critical molecular window for the early identification of individuals at risk for AMD.

Research into the etiology of AMD has long focused on the contributions of germline genetic variants. Large‐scale GWAS have identified several core susceptibility loci, including CFH (Fritsche et al. 2016) and ARMS2/HTRA1 (Gorman et al. 2024; Akagi‐Kurashige et al. 2015), establishing the dominance of complement activation and immune dysregulation in the pathogenesis of the disease. Our study highlights the critical complementary role of LOY as an acquired somatic mutation. Subgroup analyses revealed that the pathogenic association of LOY is most pronounced among individuals with high PRS. These findings suggest that germline genetic liabilities, such as polymorphisms in the complement pathway, predispose the retina to inflammatory damage by impairing immune regulatory capacity; meanwhile, the monocyte dysfunction induced by LOY serves as an active, later‐onset risk factor that further triggers local cascade pathological responses. Furthermore, the observation of a stronger LOY effect in non‐myopic individuals may reflect variations in the sensitivity of the retina to systemic pro‐inflammatory stimuli and circulating protein deposition across different ocular anatomical backgrounds (Lin et al. 2026; Lin et al. 2016).

This study possesses several notable strengths, including the utilization of a large‐scale prospective cohort from the UKB with a 15‐year follow‐up, and the integration of multi‐omic evidence spanning single‐cell transcriptomics and proteomics to establish a comprehensive evidence chain from somatic mutations to retinal structural alterations. Nevertheless, several profound limitations warrant consideration. First, while mediation analyses and proteomic trajectory modeling provide insights into the potential link between LOY and AMD, this study remains observational in nature. The lack of direct functional validation—such as through CRISPR/Cas9‐mediated gene editing or bone marrow transplantation models to interrogate candidate genes like IFI30 or CKB—precludes the definitive establishment of molecular‐level causality. Second, our analyses were restricted to the UK Biobank without replication in an independent external validation cohort (such as the All of Us research program), which limits the cross‐population robustness of our findings. Third, the UK Biobank cohort predominantly comprises individuals of European ancestry. Given the ancestral variations in genetic architecture related to the complement system and immune responses, the generalizability of the LOY‐AMD association to Asian or African populations requires further validation in diverse global cohorts. Fourth, the identification of LOY was based on the conventional mLRRY probe intensity method; although cost‐effective for large‐scale screening, it lacks the sensitivity required to capture low‐level mosaicism or complex structural variations. In future studies, incorporating pseudo‐autosomal region (PAR) phased BAF profiling could be integrated to determine whether the conclusions remain consistent. Finally, the clinical classification of AMD relied primarily on ICD codes and self‐reported diagnoses, which may mask potential heterogeneity in the LOY association between dry and neovascular AMD subtypes. Future research should prioritize the development of humanized LOY models and incorporate multicenter, multi‐ethnic validation cohorts and multi‐omics data to further elucidate the precise mechanisms by which somatic variations drive retinal degeneration.

In summary, this study identifies the significant association between LOY and the risk of age‐related ophthalmic diseases in middle‐aged men. By integrating large‐scale prospective cohort data with multi‐omic analyses, we demonstrate that LOY correlates with a pro‐inflammatory myeloid bias and triggers systemic proteomic dysregulation—marked by early deviations in plasma proteins including MMP12 and LAIR1—well before clinical manifestation. These findings establish a clear somatic mutation‐immune‐retinal axis, positioning LOY not merely as a marker of senescence but as a potential mediator of ocular‐systemic degeneration. Our results provide a robust framework for early risk stratification and highlight systemic immune modulation as a promising therapeutic frontier for preventing irreversible vision loss.

## Author Contributions

T.C. and Z.X. co‐designed the study. T.C., Z.Z., and B.S. conducted the longitudinal cohort analysis and trajectory modeling. T.C., X.D., Z.W., and S.L. performed the statistical and bioinformatics analyses. T.C., L.W., and Y.Y. developed the machine learning predictive models. T.C. drafted the initial manuscript. X.Z., J.Z., and Y.L. critically revised the manuscript for important intellectual content. All authors approved the final version.

## Funding

This work was supported by the National Key R&D Program of China (2024YFC2510805), the Shanghai Rising‐Star Program (23QA1401000), the Shanghai Oriental Talent Plan Youth Program (QNKJ2024055), and the Shanghai Hospital Development Center Foundation (SHDC12025144).

## Conflicts of Interest

The authors declare no conflicts of interest.

## Supporting information


**Figure S1:** Association Between Genetic Predisposition (AMD‐PRS) and LOY Metrics. (A, B) Scatter plots showing correlations between the AMD standard polygenic risk score (PRS) and mLRRY or LOY cell percentage, respectively. (C, D) Violin plots depicting the distribution of mLRRY and LOY cell percentage across individual PRS quintiles (Q1–Q5), with pairwise comparisons indicating no significant differences (ns).
**Figure S2:** Proteomic Landscape Associated with LOY and Incident AMD. (A–C) Volcano plots displaying the association between 2920 plasma proteins and three different mLOY indices: mLRRY, LOY status, and estimated LOY cell percentage. (D) Volcano plot showing the longitudinal association (Hazard Ratio) between plasma proteins and the risk of incident AMD. (E) Venn diagram illustrating the intersection of proteins significantly associated with the three mLOY metrics, identifying 137 core LOY‐associated proteins.
**Figure S3:** Mendelian randomization (MR) analysis of monocyte traits and age‐related macular degeneration (AMD) using the inverse variance weighted (IVW) method. Odds ratios (ORs) and 95% confidence intervals (CIs) for AMD risk in the FinnGen (Finn‐AMD) and MVP (MVP‐AMD) cohorts are shown across various monocyte categories, including counts and proportions, surface markers, morphological parameters, and overall counts. Statistically significant associations (*p* < 0.05) are highlighted in color.
**Figure S4:** Sensitivity analyses of monocyte traits on AMD risk using complementary MR methods. (A) MR‐Egger, (B) Simple mode, (C) Weighted median, and (D) Weighted mode methods evaluated across Finn‐AMD and MVP‐AMD cohorts. Error bars represent 95% CIs, and significant estimates are highlighted.
**Figure S5:** Quality control and LOY distribution in single‐cell datasets. UMAP visualization of integrated PBMC samples from the validation cohort. (B) Feature plots displaying the expression of canonical marker genes used for cell‐type annotation. (C) Density plot showing the relationship between LOY‐scores and cell proportions. (D, E) Proportional distribution of cells with absolute Y‐loss (score = 0) versus non‐LOY cells in AMD and control groups. (F) Bar chart displaying the frequency of LOY across 13 annotated choroidal cell types.
**Figure S6:** Correlation between age and LOY scores across monocyte subsets. Scatter plots with linear regression trend lines illustrating the association between chronological age and mean LOY scores in (A) overall populations, (B) CD14^+^ monocytes, (C) CD16^+^ monocytes, and (D) total monocytes, stratified by AMD and control groups.
**Figure S7:** Differential expression of LOY‐associated genes in monocytes. Violin plots comparing the expression levels of genes encoding prioritized LOY‐associated proteins in monocytes between AMD patients and controls. Statistical significance was determined using differential expression analysis within the monocyte clusters.
**Figure S8:** Cell‐type specificity of prioritized proteomic signatures. Feature plots projecting the transcriptomic expression of 29 detected LOY‐associated genes onto the PBMC UMAP space. Color intensity represents the normalized expression level, highlighting molecules with monocyte‐specific profiles such as IFI30 and CKB.
**Figure S9:** Correlation heatmap between LOY, proteins, and retinal morphometry. Heatmap displaying the Pearson correlation coefficients (β) between prioritized plasma proteins, mLOY indices, and 40 OCT‐derived retinal structural parameters. Asterisks denote statistical significance (*p < 0.05, **p < 0.01, ***p < 0.001). Retinal subfields and layers are defined according to the TABS automated segmentation algorithm.
**Figure S10:** Comparative predictive performance for incident AMD. Time‐dependent receiver operating characteristic (ROC) curves and corresponding area under the curve (AUC) values comparing the predictive performance of LOY‐associated proteins, the mLRRY metric, and the AMD polygenic risk score (AMD‐PRS) over 5‐, 10‐, and 15‐year horizons in both the training cohort (top panels) and the independent validation cohort (bottom panels).
**Figure S11:** Incremental predictive utility of mLRRY over LOY‐associated proteins for incident AMD. Time‐dependent receiver operating characteristic (ROC) curves and corresponding area under the curve (AUC) values comparing the predictive performance between LOY‐associated protein models alone (Model A, red) and models incorporating mLRRY (Model B, blue) over 5‐, 10‐, and 15‐year horizons in the training cohort (top panels) and the independent validation cohort (bottom panels).
**Figure S12:** Incremental predictive utility of mLRRY over AMD‐PRS for incident AMD. Time‐dependent receiver operating characteristic (ROC) curves and corresponding area under the curve (AUC) values comparing the predictive performance between AMD polygenic risk score models alone (Model A, green) and models incorporating mLRRY (Model B, dark blue) over 5‐, 10‐, and 15‐year horizons in the training cohort (top panels) and the independent validation cohort (bottom panels).

**Table S1:** ICD codes for ophthalmic conditions.
**Table S2:** UK Biobank data fields.
**Table S3:** Cross‐sectional association between LOY indices and ophthalmic conditions.
**Table S4:** Propensityscore matching (PSM) balance diagnostics and post‐matching baseline characteristics.
**Table S5:** Proportional hazards assumption tests based on Schoenfeld residuals for the Cox proportional hazards models.
**Table S6:** Primary care diagnostic codes and related sensitivity analyses for AMD.
**Table S7:** Longitudinal association between mLOY indices and ophthalmic conditions.
**Table S8:** Longitudinal associations between mLOY indices and incident ophthalmic conditions after propensity score matching (PSM).
**Table S9:** Subgroup and interaction analyses for incident AMD.
**Table S10:** Plasma proteins significantly associated with mLRRY.
**Table S11:** Plasma proteins significantly associated with estimated LOY cell percentage.
**Table S12:** Plasma proteins significantly associated with binary LOY status.
**Table S13:** Association between all plasma proteins and incident AMD.
**Table S14:** Intersection of LOY‐associated and AMD‐associated proteins.
**Table S15:** LOESS‐modeled temporal trajectories of prioritized proteins.
**Table S16:** Gene Ontology (GO) Enrichment Analysis of LOY‐associated proteomes.
**Table S17:** Linear association between mLOY indices and hematological parameters.
**Table S18:** Mediation analysis of the association between mLOY and incident AMD via hematological parameters.
**Table S19:** GWAS datasets for AMD and monocyte traits and corresponding Mendelian randomization analyses.
**Table S20:** Canonical marker genes for PBMC cell type annotation.
**Table S21:** Canonical marker genes for choroidal cell type annotation.
**Table S22:** Linear association between prioritized proteins and retinal morphometry.
**Table S23:** Linear association between mLOY status and retinal morphometry.

## Data Availability

The data that support the findings of this study are available from UK Biobank. Restrictions apply to the availability of these data, which were used under license for this study. Data are available from the author(s) with the permission of UK Biobank.
