# Supplementary material for: Systemic Loss of Chromosome Y Associates With Age‐Related Ophthalmic Diseases in Middle‐Aged Men: A Longitudinal Multi‐Omic Study
Source: Aging Cell. 2026 Oct 18;25(10):e70775. doi: 10.1111/acel.70775 (PMC13677818; doi:10.1111/acel.70775)
Supplement: Supplementary file 1 [file ACEL-25-e70775-s001.docx]

**Supplementary figures**

**Supplementary Figure S1. Association Between Genetic Predisposition (AMD-PRS) and LOY Metrics.**

(A–B) Scatter plots showing correlations between the AMD standard polygenic risk score (PRS) and mLRRY or LOY cell percentage, respectively. (C–D) Violin plots depicting the distribution of mLRRY and LOY cell percentage across individual PRS quintiles (Q1–Q5), with pairwise comparisons indicating no significant differences (ns).

**Supplementary Figure S2. Proteomic Landscape Associated with LOY and Incident AMD.**

(A–C) Volcano plots displaying the association between 2,920 plasma proteins and three different mLOY indices: mLRRY, LOY status, and estimated LOY cell percentage. (D) Volcano plot showing the longitudinal association (Hazard Ratio) between plasma proteins and the risk of incident AMD. (E) Venn diagram illustrating the intersection of proteins significantly associated with the three mLOY metrics, identifying 137 core LOY-associated proteins.

**Supplementary Figure S3. Mendelian randomization (MR) analysis of monocyte traits and age-related macular degeneration (AMD) using the inverse variance weighted (IVW) method.** Odds ratios (ORs) and 95% confidence intervals (CIs) for AMD risk in the FinnGen (Finn-AMD) and MVP (MVP-AMD) cohorts are shown across various monocyte categories, including counts and proportions, surface markers, morphological parameters, and overall counts. Statistically significant associations (P < 0.05) are highlighted in color.

**Supplementary Figure S4. Sensitivity analyses of monocyte traits on AMD risk using complementary MR methods.** (A) MR-Egger, (B) Simple mode, (C) Weighted median, and (D) Weighted mode methods evaluated across Finn-AMD and MVP-AMD cohorts. Error bars represent 95% CIs, and significant estimates are highlighted.

**Supplementary Figure S5. Quality Control and LOY Distribution in Single-cell Datasets.**

(A) UMAP visualization of integrated PBMC samples from the validation cohort. (B) Feature plots displaying the expression of canonical marker genes used for cell-type annotation. (C) Density plot showing the relationship between LOY-scores and cell proportions. (D–E) Proportional distribution of cells with absolute Y-loss (score = 0) versus non-LOY cells in AMD and control groups. (F) Bar chart displaying the frequency of LOY across 13 annotated choroidal cell types.

**Supplementary Figure S6. Correlation Between Age and LOY Scores Across Monocyte Subsets.**

Scatter plots with linear regression trend lines illustrating the association between chronological age and mean LOY scores in (A) overall populations, (B) CD14^+^ monocytes, (C) CD16^+^ monocytes, and (D) total monocytes, stratified by AMD and control groups.

**Supplementary Figure S7. Differential Expression of LOY-Associated Genes in Monocytes.**

Violin plots comparing the expression levels of genes encoding prioritized LOY-associated proteins in monocytes between AMD patients and controls. Statistical significance was determined using differential expression analysis within the monocyte clusters.

**Supplementary Figure S8. Cell-Type Specificity of Prioritized Proteomic Signatures.**

Feature plots projecting the transcriptomic expression of 29 detected LOY-associated genes onto the PBMC UMAP space. Color intensity represents the normalized expression level, highlighting molecules with monocyte-specific profiles such as IFI30 and CKB.

**Supplementary Figure S9. Correlation Heatmap Between LOY, Proteins, and Retinal Morphometry.**

Heatmap displaying the Pearson correlation coefficients (β) between prioritized plasma proteins, mLOY indices, and 40 OCT-derived retinal structural parameters. Asterisks denote statistical significance (*P < 0.05, **P < 0.01, ***P < 0.001). Retinal subfields and layers are defined according to the TABS automated segmentation algorithm.

**Supplementary Figure S10. Comparative Predictive Performance for Incident AMD.**

Time-dependent receiver operating characteristic (ROC) curves and corresponding area under the curve (AUC) values comparing the predictive performance of LOY-associated proteins, the mLRRY metric, and the AMD polygenic risk score (AMD-PRS) over 5-, 10-, and 15-year horizons in both the training cohort (top panels) and the independent validation cohort (bottom panels).

**Supplementary Figure S11. Incremental Predictive Utility of mLRRY over LOY-Associated Proteins for Incident AMD.**

Time-dependent receiver operating characteristic (ROC) curves and corresponding area under the curve (AUC) values comparing the predictive performance between LOY-associated protein models alone (Model A, red) and models incorporating mLRRY (Model B, blue) over 5-, 10-, and 15-year horizons in the training cohort (top panels) and the independent validation cohort (bottom panels).

**Supplementary Figure S12. Incremental Predictive Utility of mLRRY over AMD-PRS for Incident AMD.**

Time-dependent receiver operating characteristic (ROC) curves and corresponding area under the curve (AUC) values comparing the predictive performance between AMD polygenic risk score models alone (Model A, green) and models incorporating mLRRY (Model B, dark blue) over 5-, 10-, and 15-year horizons in the training cohort (top panels) and the independent validation cohort (bottom panels).
